# Amino acid signatures in the HLA class II peptide-binding region associated with protection/susceptibility to the severe West Nile Virus disease

**DOI:** 10.1371/journal.pone.0205557

**Published:** 2018-10-31

**Authors:** Constantina A. Sarri, Georgios E. Papadopoulos, Anna Papa, Athanasios Tsakris, Danai Pervanidou, Agoritsa Baka, Constantina Politis, Charalambos Billinis, Christos Hadjichristodoulou, Zissis Mamuris, MALWEST project

**Affiliations:** 1 Department of Biochemistry and Biotechnology, University of Thessaly, Larissa, Greece; 2 1st Microbiological Laboratory, Aristotle University of Thessaloniki, Thessaloniki, Greece; 3 Medical School, National and Kapodistrian University of Athens, Athens, Greece; 4 Hellenic Centre for Disease Control and Prevention (HCDCP), Athens, Greece; 5 Hellenic coordinating Hemovigilance Center of HCDCP, Athens, Greece; 6 Faculty of Veterinary Medicine, University of Thessaly, Karditsa, Greece; 7 Faculty of Medicine, University of Thessaly, Larissa, Greece; Centro Cardiologico Monzino, ITALY

## Abstract

The MHC class II region in humans is highly polymorphic. Each MHC molecule is formed by an α and a β chain, produced by different genes, creating an antigen-binding groove. In the groove there are several pockets into which antigens anchor and fit. The affinity of this fitting determines the recognition specificity of a given peptide. Here, based on our previous results about the association of MHC class II with the WNV disease, we examined the role of the binding pockets of HLA-DPA1, -DQA1 and–DRB1 in the severe form of the disease. In HLA-DQA1, variants in all pockets 1, 6 and 9 were found to be associated with either protection and/or susceptibility to neuroinvasion caused by WNV. Similarly, pockets 7, 9 and 10 in HLA-DRB1 were associated with severe disease. Protein modeling of these molecules revealed structural and functional differences among alleles with opposite roles concerning the development of the disease. Different amino acids in positions α52 and α66 (HLA-DQA1) significantly influenced the peptide binding while DYWLR/EFA combination (HLA-DRB1) was associated with neuronal damage. Further studies could help us understand the selectivity of pocket variants in order to create suitable peptides for an effective response.

## Introduction

A major part of the development of a specific immune response to a pathogen belongs to MHC class II molecules [1, 2]. MHC class II molecules are constitutively expressed by professional antigen-presenting cells (APCs), such as dendritic cells, B cells and cells of the monocyte/macrophage lineage. In addition, non-professional APCs (e.g., fibroblasts, astrocytes, epithelial cells) acquire MHC class II expression when exposed to a variety of stimuli, most potent IFN-γ, during infection, inflammation or trauma. In humans, T cells can also be induced to express class II molecules after activation [3, 4]. MHC class II molecules classically present extracellular antigens such as bacteria, parasites, and toxins that enter the host cell by endocytosis or phagocytosis, to antigen-specific CD4+ T cells. However, mechanisms of an endogenous presentation by antigen-presenting cells have been described while sufficient TCD4+ engagement is an important predictor of outcome with several viral infectious diseases (including hepatitis A and B and influenza) [5].

The MHC class II region in humans (otherwise HLA, Human Leukocyte Antigen) contains three distinct gene loci, HLA-DP, -DQ, and–DR [6]. MHC class II molecules consist of two chains, α and β. DPA, DQA and DRA genes code for chain α, while DPB, DQB and DRB genes code for chain β. Except for DRA, which is dimorphic with just one substitution at the intra-cytoplasmic position 227, the MHC class II locus is highly polymorphic. Up to date 45 DPA1 alleles, 78 DQA1 alleles, 828 DPB1 alleles, 1,079 DQB alleles and 2,058 DRB1 alleles have been identified [7, 8, 9]. The high polymorphism observed is mainly located in exon 2 of these molecules that codes for the peptide-binding region (PBR).

**Fig 1:**
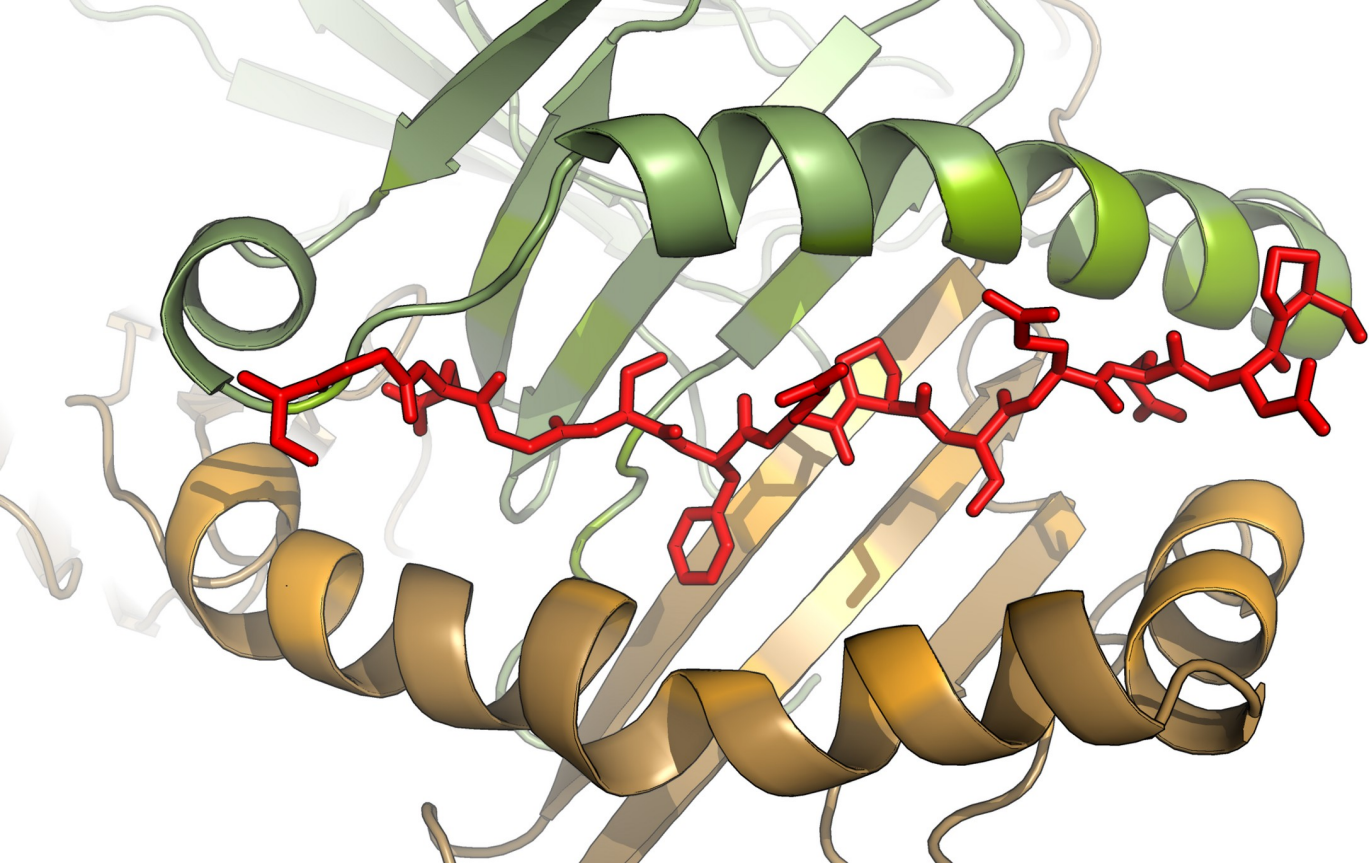
MHC class II molecules are heterodimers consisting of α (green) and β (orange) chains. The antigen binding groove is formed by two alpha-helices above a beta-sheet floor. Antigens (red) fit in the cleft and stabilized by electrostatic interactions and/or hydrogen bonds to anchor peptides of the groove (regions with light shades). The structure being illustrated is based on the structure with PDB ID: 2NNA [12].

The PBR resides in a peptide antigen-binding groove, formed by both α and β chains. In the cleft, MHC molecules feature binding pockets into which anchoring peptides of the antigen can fit. The fitting is stabilized by hydrogen bonds and ion bridges between the side chains of α and β chains of the groove and along the backbone of the peptide (Fig 1). The groove of class II molecules is open, allowing the binding of a wide range of peptides; typically 12–26 amino acids long. The peptide segments that bind to groove are nonamers while their N and C termini may extend beyond the ends of the groove [10]. The flanking residues can increase the binding affinity to class II MHC molecules or can also be used directly by T cell receptor as contact residues [11].

The four major binding pockets are P1, P4, P6 and P9 while P7 [11, 13] and P10 can also influence the peptide binding selectivity [14]. These pockets vary between MHC molecules that are encoded by different alleles (a pocket variant is considered any combination of amino acid in the binding pocket), having a narrower or wider specificity for different residues and thus different affinity of binding for the given peptides [15]. So, it becomes evident that the array of PBR variation determines the range of recognizable pathogen-derived antigens.

In our previous study, we examined the polymorphism of three MHC class II genes, *HLA-DPA1*, *HLA-DQA1* and *HLA-DRB1*, in a cohort of WNV cases and a control group of the Greek population in order to investigate the contribution of MHC class II background in the infection and outcome of the WNV disease. Alleles that indicate protection against or susceptibility to WNV infection as well as alleles that may protect from neuroinvasion where identified in all three loci [16]. Here, we focused on the involvement of MHC class II in neuroinvasion, by targeting the PBR. The aim of this study is to use a) the differentiation of the frequency of the pocket variants that belong to the genes under study, between the mild and severe clinical symptoms of WNV disease and b) the structural analysis of the pocket variants that are either protective or susceptible against neuroinvasion, in order to better understand the role of each pocket in WNV recognition and suggest a model of how these amino acid changes could influence the outcome of the disease.

## Materials and methods

### Selection of the positions for the analysis

In this study, we retrieved the results from our previous work [16] and focused on residues that form binding pockets P1, P4, P6, P7, P9 and P10. In the MHC class II heterodimer, chains α and β, both contribute to the formation of pockets P1, P6, P9 and P10 while chain β forms alone P4 and P7. In particular, positions α- 7, 9, 24, 31, 32 43, and 52 (P1), 11, 62, 65, 66 and 69 (P6), 68, 69, 72, 73 and 76 (P9) and β- 85, 86, 89 and 90 (P1), 13, 26, 28, 70, 74 and 78 (P4), 11, and 30 (P6), 28, 47, 61, 67 and 71 (P7), 9, 37 and 57 (P9), 56, 57 and 60 (P10) were analyzed [13, 14]. Position α76 belongs in P10 but was not included in the analysis due to monomorphism [17].

### Populations under study

A total of 105 confirmed WNV cases were used in our study. Sixty-eight individuals were reported with West Nile Neuroinvasive Disease (WNND) while thirty-seven individuals developed only mild symptoms and are referred to as West Nile Fever (WNF) cases [16]. Considering other risk factors in the populations under study, no sufficient information for immunosuppression of cases was available. Additionally, cases in both groups had similar age (age in years, mean ± s.d.: WNND 67±16.18, WNF 63±16). WNND was associated with men [16] as described in the literature [18].

### Statistical analysis

The association of each pocket or combination of pockets with WNV disease outcome was examined with pairwise comparison using the z test (XLSTAT 2014.5.03). P<0.05 was considered statistically significant in all tests. The analyses for association between WNV cases sub-groups and amino acid residues were performed using the χ^2^ test.

### Modeling

The following protocol has been followed in order to construct models of complexes DQA1*01:02-DQB1*03:01, DQA1*03:01-DQB1*03:01, DRA1*01-DRB1*11:04 and DRA1*01-DRB1*14:04 to evaluate the structural and functional consequences of allele sequence differences. The crystal structures of PDB entry code: 2NNA [12] and PDB entry code: 2WBJ [19] have been used as starting conformations for molecular dynamics (MD) simulations in explicit water. DQA1*01:02 and DQA1*03:01 were created after the following amino acid alterations in the 2NNA structure; DQA1*01:02: 9 Tyr>Cys, 15 Ser>Phe, 23 Ser>Thr, 31 Glu>Gln, 42 Val>Ala, 44 Gln>Arg, 45 Leu>Trp, 47 Leu>Glu, 49 Arg>Ser, 50 Arg>Lys, 52 Gly>Arg, 53 Gly>Arg, 58 Phe>Gly, 61 Thr>Arg, 63 Ile>Met, 66 Leu>Ala, 73 Val>Met and 77 Ser>Tyr, DQA1*03:01: 41 Thr>Val and 42 Val>Ala. Similarly, DRB1*11:04 and DRB1*14:04 were created after the following amino acid alterations in the 2WBJ structure; DRB1*11:04: 9 Trp>Glu, 10 Gln>Tyr, 11 Pro>Ser, 12 Lys>Thr, 13 Arg>Ser, 37 Ser>Tyr, 58 Ala>Glu, 67 Ile>Phe, 70 Gln>Asp and 71 Ala>Arg, DRB1*14:04: 9 Trp>Glu, 10 Gln>Tyr, 11 Pro>Ser, 12 Lys>Thr, 13 Arg>G, 16 His>Tyr, 32 Tyr>His, 37 Ser>F, 47 Phe>Tyr, 57 Asp>Ala, 60 Tyr>His, 67 Ile>Leu, 70 Gln>Arg, 71 Ala>Arg and 74 Ala>Glu. Disulphide bonds have been added as indicated in the PDBs.

The protonation states were first determined with PROPKA [20, 21] and then adjusted accordingly. The solvated systems (protein complex DQA1*01:02-DQB1*03:01, 12777 waters, 52 Na+ and 88 Cl^-^ ions, DQA1*03:01-DQB1*03:01, 12353 51 Na+ and 86 Cl^-^ ions, DRA1*01-DRB1*11:04, 14043 water molecules, 61 Na+ and 101 Cl^-^ ions, DRA1*01-DRB1*14:04, 14043 water molecules, 63 Na+ 103 Cl^-^ ions) were first energy minimized and then subjected to 15000 steps of NPT MD simulation at 310K and 1 Atm with the Particle Mesh Ewald (PME) algorithm for handling electrostatics in order to adjust the simulation cell dimensions. The resulting systems have been then simulated in the NVT ensemble for 3 ns at 310K. The final systems (the last frame of the MD trajectories) were subjected to energy minimization. The obtained minimized protein conformations were used for further structural analysis and comparisons. We should note that during simulation of heterodimer DRA1*01-DRB1*14:04, an Asp existed in position 57. This residue was later mutated to an Ala. After the change to an Ala, a cycle of minimization for 10000 steps followed. The MD program NAMD [22] and the CHARMM force field parameter and topology for proteins and nucleic acids [23] were used for the simulations. RMSD average and RMSD per-residue were calculated (S1 Fig). Molecular model illustrations were rendered using PyMOL (The PyMOL Molecular Graphics System, Schrödinger, LLC). Hydrogen bonds detection was based on the default criteria of PyMOL. Evaluation of salt bridges was performed by ESBRI [24].

## Results

**Fig 2:**
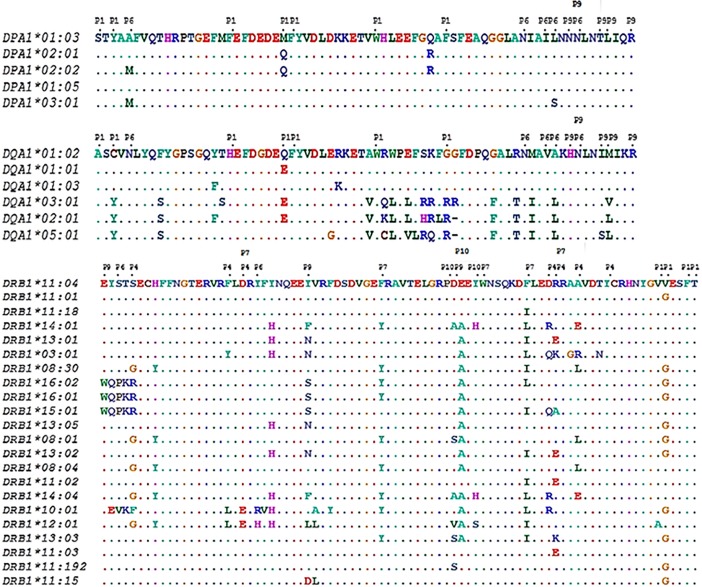
The amino acid sequence of exon 2 (partial) of the alleles that were identified in HLA-DPA1, -DQA1 and DRB1 in our WNV cases. The residues that form pockets P1, P4, P6, P7, P9 and P10 have been noted accordingly. Positions that have the same amino acid with the first sequence in other alleles are shown with dots.

The amino acid sequence of the previously identified alleles in our cohort under study is shown in Fig 2. In HLA-DPA1, five residues of a total of 82 were polymorphic (6.1%) in the five alleles identified. In HLA-DQA1, 18 positions were variable (22%) in a total of six alleles while in 22 DRB1 alleles, 26 polymorphic positions were found in a total of 89 residues (29.2%) that are coded from exon 2. Although polymorphisms were found throughout exon 2, the PBR was a ‘hot-spot’ as it was expected. Focusing on the selected pockets (Materials & Methods), three residues were variable out of 16 in HLA-DPA1’s PBR (~19%). In HLA-DQA1 the variability of the PBR was two times higher than HLA-DPA1 (6 of 16 residues, 37.5%). Finally, HLA-DRB1 showed an extreme percentage of polymorphism (76.2%) where only 5 residues were conserved in the 22 alleles identified. However, amino acids with similar biochemical properties that were found in these variable sites could have little effect in the functional polymorphism of the molecule.

*HLA-DPA1* had the least polymorphic pockets. P1 was dimorphic while P6 had three variants; SYFMFWF and SYFQFWF and ANILN, MNILN and MNISN were found in P1 and P6 respectively. P9 was monomorphic (NNTLR) (Table 1, Fig 2). These variants were found to be combined in four amino acid sequences (Fig 2). SYFMFWF (P1) and ANILN (P6) were abundant in both groups (Table 1). Variant MNISN (P6) was found only in WNND group, with low frequency (Table 1).

**Table 1:** The pocket variants and their frequency found in HLA-DPA1, -DQA1 and -DRB1 in both WNND and WNF case groups. The pocket in which each variant belongs is shown on the left of the variant. The variants found exclusively in one of the groups are shown in italics (in the WNND/WNF columns).

	**WNND**	**WNF**	p-value		WNND	WNF	p-value		WNND	WNF	p-value		WNND	WNF	p-value
**DPA1**				**DRB1**				**DRB1**				**DRB1**			
SYFMFWF(1)	0,846	0,851		VVFT(1)	0,579	0,546	0,6708	SH(6)	*0*,*044*	*0*	0,0887	EYS(9)	0,036	0,016	0,4448
SYFQFWF(1)	0,154	0,149		VGFT(1)	0,377	0,454	0,3168	DFWFR(7)	0,351	0,329	0,7674	ELV(9)	*0*,*044*	*0*	0,0887
ANILN(6)	0,949	0,955		AVFT(1)	*0*,*044*	*0*	0,0887	DFWIR(7)	*0*	*0*,*016*	0,1794	PDY(10)	0,843	0,973	**0,0079**
MNILN(6)	0,044	0,045		SFDDRAY(4)	0,351	0,345	0,9360	DYWLR(7)	0,114	0,016	**0,0200**	PAH(10)	*0*,*052*	*0*	0,0636
MNISN(6)	*0*,*07*	*0*		SFDRREY(4)	*0*,*052*	*0*	0,0636	DFWIE(7)	0,062	0,125	0,1492	PSY(10)	0,036	0,016	0,4448
NNTLR(9)	1	1		SFDDEAY(4)	0,062	0,141	0,0792	DFWLK(7)	0,079	0,094	0,7307	PAY(10)	0,027	0,016	0,6396
**DQA1**				SYDQKRY(4)	0,079	0,094	0,7307	DYWIR(7)	*0*	*0*,*016*	0,1794	PVY(10)	*0*,*044*	*0*	0,0887
ACHQFWG(1)	0,583	0,548		GFDDRLY(4)	0,044	0,032	0,6947	DFWIA(7)	0,15	0,126	0,6601				
ACHEFWG(1)	0,287	0,194		RFDDRAY(4)	0,149	0,188	0,5006	EYWLR(7)	0,017	0,063	0,1039				
AYHEFWR(1)	0,113	0,226		RFDQAAY(4)	0,15	0,126	0,6601	EFWIR(7)	*0*,*044*	*0*	0,0887				
AYHQFWR(1)	0,017	0,032		GFDRREY(4)	0,027	0,016	0,6396	DFWFE(7)	*0*	*0*,*016*	0,1794				
NNVAN(6)	0,87	0,742		FLERRAY(4)	0,017	0,063	0,1039	DYWFR(7)	0,158	0,204	0,4395				
NNVLN(6)	0,13	0,258		GLEDRAY(4)	*0*,*044*	*0*	0,0887	DYWIK(7)	*0*,*027*	*0*	0,1848				
HNIMR(9)	0,87	0,742		SFDDKAY(4)	*0*,*027*	*0*	0,1848	EYD(9)	0,386	0,471	0,2712				
HNIVR(9)	0,087	0,161		SY(6)	0,642	0,628	0,8526	EFA(9)	0,079	0,016	**0,0376**				
HNILR(9)	0,026	0,065		PY(6)	0,299	0,314	0,8352	END(9)	0,158	0,188	0,6094				
HNSLR(9)	0,017	0,032		VR(6)	0,017	0,063	0,1039	WSD(9)	0,299	0,314	0,8352				

*Pairwise comparisons were performed using z test. Comparisons with p-value <0.05 were considered statistically significant.

Higher PBR variation was observed in the 6 DQA1 alleles found in the population studied. In P1, 4 variants were identified; ACHQFWG, ACHEFWG, AYHEFWR and AYHQFWR. Analogously, 4 variants were found in P9; HNIMR, HNIVR, HNILR and HNSLR. P6 was dimorphic; NNVAN and NNVLN (Table 1). ACHQFWG of P1 was the most frequent variant identified in both WNND and WNF. The frequency of AYHEFWR and AYHQFWR was two times higher in WNF compared to WNND. In P6 and P9, variants NNVAN and HNIMR were found with higher frequency (Table 1). Five different combinations were observed. As shown in Fig 2, DQA1*01:02 and DQA*01:03 differ in two amino acids that are not included in the PBR.

Twenty-two variant combinations were observed in 22 alleles found in the case of DRB1s (Fig 2). Binding pockets P4 and P7 showed higher polymorphism (11 and 12 variants respectively) while P1 and P6 were more conserved (3 and 4 variants respectively) amongst the proteins. Six variants were found in P9 and five in P10. The most frequent variants in both cohorts in all pockets were VVFT (P1), SFDDRAY (P4), SY (P6), DFWFR (P7), EYD (P9) and PDY (P10). The combination of these variants was found in DRB1*11:04. AVFT (P1), SFDRREY, GLEDRAY and SFDDKAY (P4), SH (P6), EFWIR and DYWIK (P7), ELV (P9) and PVY and PAH (P10) were found only in the WNND group. On the contrary, DFWIR, DYWIR and DFWFE of P7 were found only in WNF (Table 1).

### PBR pockets associated with neuroinvasion

**Fig 3:**
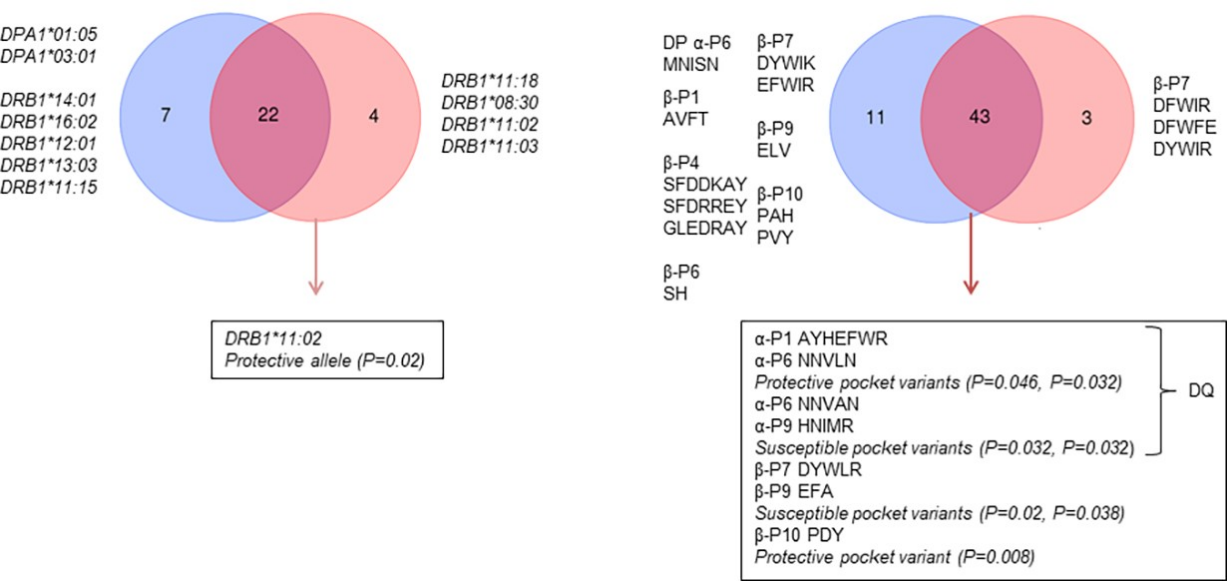
The distribution of alleles and pocket variants in WNND (blue) and WNF (light red). Next to each group are listed the alleles that were unique for the group. In the boxes below, the alleles/pocket variants that were associated positively or negatively with neuroinvasion are shown.

Concerning the PBR of *HLA-DQA*, all pockets seem to have a specific role for binding WNV peptides. Specifically, in P1, the frequency of AYHEFWR was two times higher in the WNF group compared with WNND patients (P = 0.046). P6 variants were found in inverse proportion in WNND and WNF cohorts; NNVLN could have a protective role against neuroinvasion (P = 0.032). In P9, HNIMR was carried by the majority of both cases and controls. However, it was found with significantly lower frequency in WNF cases compared to WNND (P = 0.032) (Fig 3).

In *HLA-DRB1*, ten pocket variants (AVFT in P1, SFDRREY, GLEDRAY and SFDDKAY in P4, SH in P6, EFWIR and DYWIK in P7, ELV in P9, PAY and PVS in P10) were implicated in neuroinvasion as they were present only in WNND individuals. DYWIR and DFWFE in P7 were found in WNF but not in WNND. Nonetheless, their low frequency was deterrent in order to characterize them as protective against neuroinvasion. DYWLR was found in 11.4% of WNND but in only 1.6% of WNF (P = 0.02). Similarly, EFA was observed in 7.9% of WNND in comparison to 1.6% in WNF (P = 0.04). Conversely, PDY, was found in the overwhelming majority of WNF (97.3%) (WNND vs WNF, P = 0.008). VVFT/SFDDEAY/SY/DFWIE/EYD/PDY (DRB1*11:02) could be involved in the protection against neuroinvasion as none of the cases bearing them developed WNND (P = 0.02) (Fig 3).

### Groove structure and protein modeling

#### The case of *HLA-DQA1*

**Pocket 1.** Susceptible ACHQFWG (data not shown, derived from the comparison of WNV cases *vs* our previous control group) and resistant AYHEFWR differ in positions *α*9, *α*31 and *α*52. *α*9-Tyr and *α*52-Arg of AYHEFWR are more spacious than *α*9-Cys and *α*52-Gly of ACHQFWG. Specifically, *α*52 Gly>Arg increases the volume of this position 3 times. Position *α*31 has similar volumes in these variants. In ACHQFWG no amino acids are charged. On the contrary, *α*31-Glu is negatively charged and *α*52-Arg is positively charged (Table 2). The existence of bulkier amino acids creates a shallow groove that may not favor a voluminous anchor peptide residue. AYHEFWR has oppositely charged residues that could affect the binding selectivity of the antigenic peptides. However, charge could also increase the immune response against WNV by increasing the binding affinity of specific epitopes.

**Table 2:** List of WNV neuroinvasion-associated amino acid residues in HLA-DQA1 and their properties [25]. P values in bold indicate significance.

Amino acid	Amino acid variants	Pocket	Properties	Volume (Α^3^)	Comparison	WNND vs WNF
position						p-value
9	Cysteine (C)	P1	Hydrophobic	108.5	C vs Y	**0.0486**
	Tyrosine (Y)		Partially hydrophobic	193.6		
31	Glutamine (Q)	P1	Polar	143.8	Q vs E	**0.0207**
	Glutamic acid (E)		Charged (-)	138.4		
52	Glycine (G)	P1	Non-polar	60.1	G vs R	**0.0001**
	Arginine (R)		Charged (+)	173.4		
66	Alanine (A)	P4	Non-polar	88.6	A vs L	**0.0001**
	Leucine (L)		Hydrophobic	166.7		
73	Methionine (M)	P9	Hydrophobic	162.9	M vs others	**0.0001**
	Leucine (L)		Hydrophobic	166.7	M vs V	0.0902
	Valine (V)		Hydrophobic	140	M vs L	**0.0433**
					V vs L	1.0000

*Pairwise comparisons were performed using x^2^ test. Comparisons with p-value <0.05 were considered statistically significant.

**Pocket 4.** No difference in charge is observed between NNVAN and NNVLN. However, *α*66 Ala>Leu increases the volume by two times in this position of the pocket. The reverse behavior of these two pockets, NNVAN being susceptible and NNVLN being resistant to neuroinvasion, could be explained by the different volume preferences of anchor peptides they present (Table 2). NNVLN was found in combination only with.

AYHEFWR that also was associated with protection against neuronic damage.

**Pocket 9.** HNIMR was associated with neuronic susceptibility. However, most of the P9 variants observed share analogous charge and volume properties. HNIMR was found in combination with ACHEFWG and ACHQFWG. On the contrary, resistant AYHEFWR (neuroinvasion) was found in combination with both HNILR and HNIVR. The abovementioned P9 variants differ at *α*73 (Met>Leu>Val). These substitutions are non-polar and have similar volume (Table 2).

**Fig 4:**
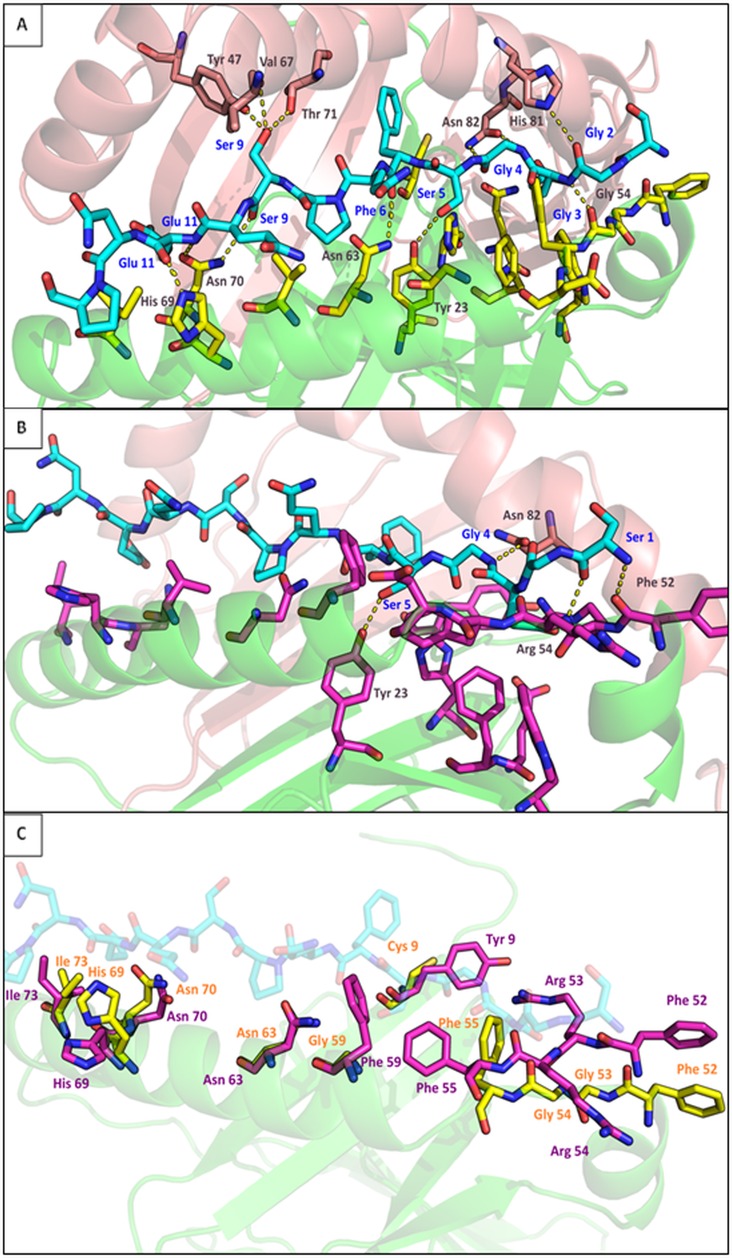
Graphical representation of DQA1/DQB1 molecules. α chain is shown in red and β chain in green. In the center of the groove, the peptide (chain C from PDB ID: 2NNA) is shown in cyan sticks. The residues of the heterodimer that are in the neighbor of the peptide are shown in CPK colored sticks (DQA1*01:02 in yellow and DQA1*03:01 in magenta). Amino acids from chain β that form H-bonds are shown in salmon. Hydrogen bonds are shown in yellow dotted lines. A. Heterodimer DQA1*01:02-DQB1*03:01. B. Heterodimer DQA1*03:01-DQB1*03:01. All neighbor residues to the peptide are shown (in radius 4 Å). Only the amino acids that form hydrogen bonds are labeled. C. Selected residues that have different orientation and/or show different interaction with the peptide between the two structures (residues 52, 55, 63, 69, 70 and 73). Variable residues of the alleles that were found in close proximity to the peptide are also shown (positions 9, 53, 54 and 59).

Protein modeling: DQA1*01:02/03:01 –DQB1*03:01. We constructed the structural models of DQA1*01:02-DQB1*03:01 (susceptible pocket variants) and DQA1*03:01-DQB1*03:01 (resistant pocket variants) based on the abovementioned association results. RMSD results (S1 Fig) were not used since no systematic discrimination of the alleles was observed. Selecting the residues that are located in a radius of 4 Å from the peptide in each molecule, we observe that several amino acids were shifted in relation to the peptide. All residues that were found in close proximity to the peptide were evaluated, even if present in only one of the model structures. Ile 73, His 69, Asn70, Asn 63, Val 66, Trp 44, Phe 55 and Asp 56 have different orientation in DQA1*01:02 and DQA1*03:01 alleles (Fig 4C). This differentiation could affect the size of the peptide’s side chain that could anchor at these residues. In addition, it could change the stability of the peptide’s binding to the groove. Indeed, Asn 63 (found in the neighbor of the peptide only in DQA1*01:02) and His 69 form two hydrogen bonds with Phe 6 and Glu 11 of the peptide in DQA1*01:02 but not in DQA1*03:01, respectively (Fig 4A, Table 3). Similarly, Asn 70 in DQA1*01:02 forms two hydrogen bonds with Ser 9 and Glu 11 of the peptide, stabilizing its binding in the groove (Fig 4A, Table 3). Ile 73, Val 66, Trp 44 and Phe 55 are located deeper in the cleft, thus their orientation did not affect the binding of this peptide (data not shown). Tyr 23 has a slight difference in orientation between the two molecules. However, in both molecules, Tyr 23 forms H-bonds. Specifically, in Tyr 23 OH is in suitable proximity to the peptide’s Ser 5 OG to form a hydrogen bond in both cases (Fig 4A and 4B, Table 3). Phe 52 showed no change in orientation between DQA1*01:02 and DQA1*03:01; however, its setting in the groove is very different (Fig 4C). A hydrogen bond is formed (Ser 1 of the peptide) only in DQA1*03:01 (Fig 4B, Table 3). Nevertheless, Phe 52 in DQA1*03:01 could also possibly block the binding of a peptide with bulky flanking N terminus.

**Table 3:** All the hydrogen bonds and the salt bridges between DQA/DQB chains and the peptide in both structures. Columns ‘Molecule 1’/‘Residue’ 1 list the α or β chain of each DQ complex (column ‘Molecule 1’) and the residues that interact with the peptide (column Residue 1). Column ‘Molecule 2’ refers always to the peptide. In column ‘Residue 2’ the peptide’s residues that form H-bonds and salt bridges with any DQ-complex amino acid are listed. The atoms involved are shown.

H-bonds				
	Molecule 1	Molecule 2	Residue 1	Residue 2
DQA1*01:02-DQB1*03:01	DQA1*01:02	peptide	OH Tyr 23	OG Ser 5
	DQA1*01:02	peptide	O Gly 54	N Glu 3
	DQA1*01:02	peptide	ND2 Asn 63	O Phe 6
	DQA1*01:02	peptide	NE2 His 69	O Glu 11
	DQA1*01:02	peptide	OD1 Asn 70	N Glu 11
	DQA1*01:02	peptide	ND2 Asn 70	O Ser 9
	DQB1*03:01	peptide	OH Tyr 47	OG Ser 9
	DQB1*03:01	peptide	O Val 67	OG Ser 9
	DQB1*03:01	peptide	OG1 Thr 71	OG Ser 9
	DQB1*03:01	peptide	NE2 His 81	O Gly 2
	DQB1*03:01	peptide	OD1 Asn 82	N Gly 4
	DQB1*03:01	peptide	ND2 Asn 82	O Gly 4
DQA1*03:01-DQB1*03:01	DQA1*03:01	peptide	OH Tyr 23	OG Ser 5
	DQA1*03:01	peptide	O Phe 52	N Ser 1
	DQA1*03:01	peptide	N Arg 54	O Ser 1
	DQB1*03:01	peptide	OD1 Asn 82	N Gly 4
**Salt bridges**				
DQA1*03:01-DQB1*03:01	DQA1*03:01	peptide	NH1 Arg 53	OE Glu 3
	DQA1*03:01	peptide	NH2 Arg 53	OE Glu 3

The different conformation observed is a result of the polymorphic sites between these alleles. Four positions were found to be occupied by different amino acids in the ray of the peptide used. Position 9 is located on the floor of the cleft while position 59 on the a-helix. However, none of the residues in these positions contributed in binding this peptide. On the other hand, amino acids in position 54 form H-bonds between different anchor residues of the peptide. DQA1*01:02 (Gly 54) forms a bond with Glu 3 (Fig 4A, Table 3) while DQA1*03:01 (Arg 54) with Ser 1 of the peptide (Fig 4B, Table 3). Arg 53 in DQA1*03:01 instead of Gly, creates two ion bonds with the peptide (Glu 3) (Table 3). These are the only salt bridges found between the alleles studied and the peptide used.

Although, the sequence of DQB in our cases is unknown and both DQA alleles have been simulated in complex with DQB1*03:01, it is noteworthy that β chain is involved in peptide fitting in disparate ways. In DQA1*01:02-DQB1*03:01 heterodimer, DQB1*03:01 forms six H bonds throughout the peptide. Both chains contribute equally in the binding of this peptide, forming 12 bonds along its length, sustaining the peptide’s anchor residues in the groove (Fig 4A, Table 3). On the contrary, heterodimer DQA1*03:01-DQB1*03:01 forms four H-bonds (DQB*03:01 forms a bond with Gly 4) and two salt bridges with the peptide (Table 3). These interactions are concentrated near one of the openings of the groove without any anchoring support in the other opening (Fig 4B).

#### Comparison between susceptible and resistant *HLA-DRB1*

**Pocket 7.** DYWLR’s frequency in WNND was seven times higher than in the WNF group. Comparison of the frequency of the amino acids found at positions *β*- *28*, *47*, *61*, *67* and *71* between WNND and WNF did not reveal any preference or association with either of the groups. Most of the amino acid substitutions have similar volumes. Some charge changes have been observed but not associated with the outcome of the disease (Table 4).

**Table 4:** List of WNV neuroinvasion-associated amino acid residues in HLA-DRB1 and their properties [25].

Amino acid	Amino acid variants	Pocket	Properties	Volume (A3)	Comparison	WNND vs WNF
position						p-value
28	Aspartic acid (D)	P7	Charged (-)	111.1	D vs E	1.0000
	Glutamic acid (E)		Charged (-)	138.4		
47	Phenylalanine (F)	P7	Hydrophobic	189.9	F vs Y	0.8665
	Tyrosine (Y)		Partially hydrophobic	193.6		
67	Phenylalanine (F)	P7	Hydrophobic	189.9	L vs F	0.5406
	Isoleucine (I)		Hydrophobic	166.7	L vs I	0.8166
	Leucine (L)		Hydrophobic	166.7	L vs all	0.5599
71	Arginine (R)	P7	Charged (+)	173.4	R vs E	0.1064
	Glutamic acid (E)		Charged (-)	138.4	R vs K	1.0000
	Lysine (K)		Charged (+)	168.6	R vs A	1.0000
	Alanine (A)		Non-polar	88.6	R vs all	0.6205
9	Glutamic acid (E)	P9	Charged (-)	138.4	E vs W	0.8649
	Tryptophan (W)		Non-polar	227.8		
37	Phenylalanine (F)	P9	Hydrophobic	189.9	F vs Y	0.0862
	Serine (S)		Polar	89	F vs N	0.1238
	Tyrosine (Y)		Partially hydrophobic	193.6	F vs S	0.1442
	Asparagine (N)		Polar	114.1	F vs L	1.000
	Leucine (L)		Hydrophobic	166.7	F vs all	0.0959
57	Alanine (A)	P9, P10	Non-polar	88.6	A vs D	0.0911
	Aspartic acid (D)		Charged (-)	111.1	A vs S	1.000
	Serine (S)		Polar	89	A vs V	1.000
	Valine (V)		Hydrophobic	140	A vs all	0.0959
60	Tyrosine (Y)	P10	Partially hydrophobic	193.6	Y vs H	1.000
	Histidine (H)		Polar	153.2	Y vs S	0.1596
	Serine (S)		Polar	89	Y vs all	0.1583

*Pairwise comparisons were performed using x^2^ test. Comparisons with p-value <0.05 were considered statistically significant.

**Pocket 9.** P9 variant, EFA, was associated with increased danger of CNS involvement. Again, other variants in this pocket have the same properties as EFA (Table 4). As in the case of DYWLR, no association was observed between variants with different characteristics.

**Pocket 10.** In P10, PDY was associated with protection against WNV neuroinvasion. This variant was found in 97.3% of the WNF patients. Variability was observed in positions *β*57 and *β*60 while *β*56 was monomorphic. *β*57 Asp and *β*60 Tyr create a more shallow pocket than the other variants. Furthermore, Asp is the only charged amino acid in both polymorphic positions of the pocket (Table 4). This could influence the binding selectivity of P10 and thus limit WNV neuroinvasion. However, no association was observed when focusing on each position separately. It seems that the combination of *β*57-Asp and *β*60-Tyr imparts the protective character of the variant rather than a specific amino acid.

Protein modeling: DRA1*01-DRB1*11:04/DRB1*14:04. Based on the pocket variants that showed different distribution between WNND and WNF groups with statistically significant association with either protection or susceptibility to neuronic damage, we constructed the structural models of DRA1*01-DRB1*11:04 (protective pocket variant) and DRA1*01-DRB1*14:04 (susceptible pocket variants). RMSD results (S1 Fig) were not used since no systematic discrimination of the alleles was observed. DRB1*14:04 was chosen because it has both DYWLR and EFA variants that were associated with neuroinvasion. During simulation of heterodimer DRA1*01-DRB1*14:04, a mutation Asp>Ala in position 57 was performed in order to see if any difference occurred that could explain the protective role of PDY. DRA1*01-DRB1*11:04 selection was based on the existence of PDY and also its frequency; it was the most frequent allele in both groups.

In DRA1*01-DRB1*11:04 fourteen residues in the neighbor of the peptide were found (4 Å). These residues are Asp 28, Tyr 30, Asp 57, Ty60, Trp 61, Gln 64, Phe 67, Asp 70, Arg 71, Ala 74, Thr 77, Tyr 78, His 81 and Asn 82. In DRA1*01– DRB1*14:04 ten amino acids in the peptide’s neighborhood were found. These residues are Phe 26, Asp28, Pro56, Ty60, Ile 67, Glu 71, Thr 77, Tyr 78, His 81 and Asn 82. Again, in order to evaluate the differences in the interacting residues between the two molecules, we took into account all the positions where at least one of the allele’s amino acid was in the immediate vicinity of the peptide (for example position 56 was not found to participate in any interaction with the peptide in DRA1*01-DRB1*11:04 but it was in interacting distance in DRA1*01-DRB1*14:04). However, since we are interested only in pockets 7, 9 and 10 the respective residues were selected for further evaluation.

**Fig 5:**
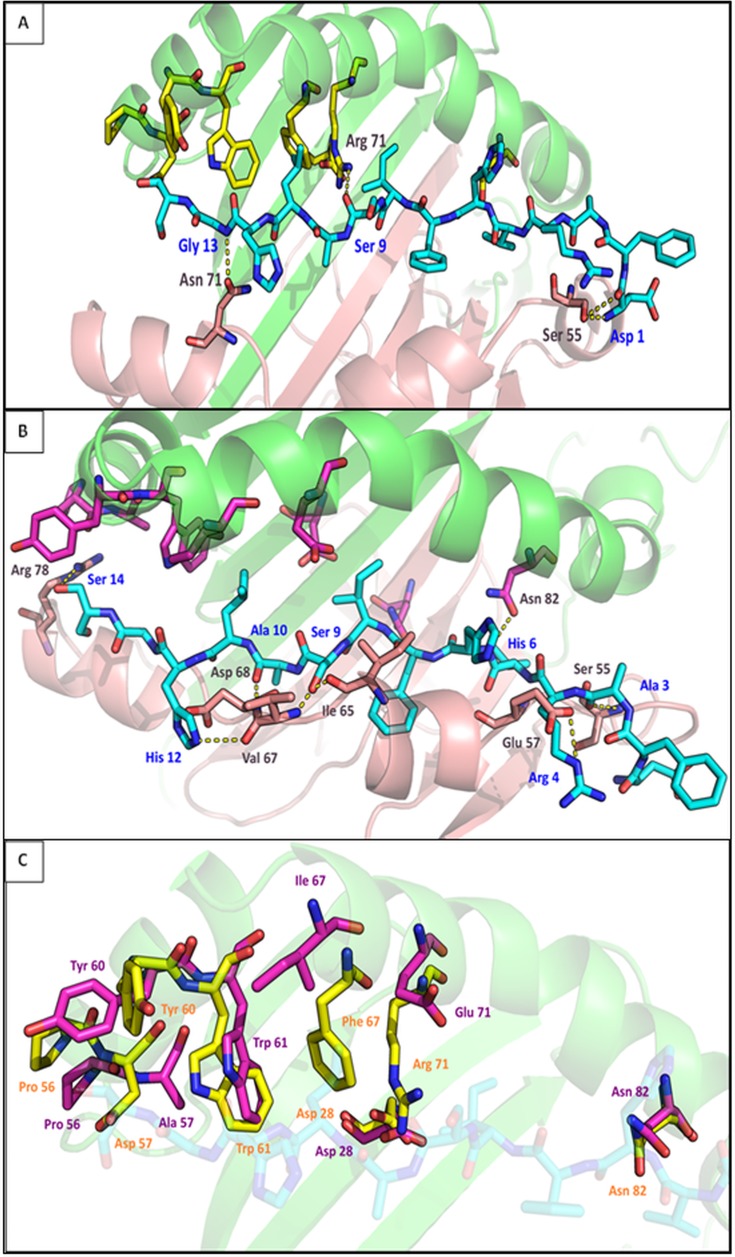
Graphical representation of DRA1/DRB1 molecules. α chain is shown in red and β chain in green. In the center of the groove, the peptide (chain D from PDB ID: 2WBJ) is shown in cyan sticks. The residues of the heterodimer that are in the neighbor of the peptide and belong in P7, P9 and P10 are shown in CPK colored sticks (DRB1*11:04 in yellow and DRB1*14:04 in magenta). Amino acids from chain α that form H-bonds are shown in salmon. Hydrogen bonds are shown in yellow dotted lines. A. Heterodimer DRA1*01– DRB1*11:04. B. Heterodimer DRA1*01– DRB1*14:04. All neighbor residues to the peptide are shown (in radius 4 Å). Only the amino acids that form hydrogen bonds are labeled. C. Selected residues that have different orientation and/or show different interaction with the peptide between the two structures (residues 28, 56, 60, 61 and 82). Variable residues of the alleles that were found in close proximity to the peptide are also shown (positions 57, 67 and 71).

Four residues on P7 are in close proximity to the peptide. A little change in orientation of Trp 61, resulted in the removal of this residue from the neighborhood of the peptide in DRB1*14:01. Phe 67 of DRB1*11:04 is bulkier than Ile 67 of DRB1*14:04 (Fig 5C). This could prevent the binding of a peptide with spacious side chains. In this case, the peptide’s Leu 11 seems that cannot fit since Phe 67 (Fig 5A) narrows the opening of the cleft. Finally, Arg 71 of DRB1*11:04 forms a hydrogen bond with the peptide (Fig 5A, Table 5). On the other hand, Glu 71 of DRB1*14:04 is very small and thus in a greater distance from the peptide (Fig 5C). Concerning P9, Asp 57 and Ala 57 did not show any difference in capacity of the space of the groove or the electrostatic interactions with the peptide. Residues that form pocket 10 are located in the opening of the groove. Since no flanking regions are available from the crystal structure we did not expect to find any interactions. However, we observed that Tyr 60 has a much differentiated orientation/position in the a-helix that could affect either the size of the accepted peptide residue or the interaction with the TCR after the binding. In DRB1*11:04, Tyr 60 has turned into the groove while in DRB1*14:04 is orientated towards the opening (Fig 5C).

**Table 5:** The hydrogen bonds and the salt bridges between DRA/DRB chains and the peptide in both structures. Columns ‘Molecule 1’/‘Residue’ 1 list the α or β chain of each DR complex (column ‘Molecule 1’) and the residues that interact with the peptide (column Residue 1). Column ‘Molecule 2’ refers always to the peptide. In column ‘Residue 2’ the peptide’s residues that form H-bonds and salt bridges with any DR-complex amino acid are listed. The atoms involved are shown.

H-bonds				
	Molecule 1	Molecule 2	Residue 1	Residue 2
DRA1*01/	DRB1*11:04	peptide	NH1 Arg 71	O Ser 9
DRB1*11:04	DRA1*01	peptide	OG Ser 55	O Asp 1
	DRA1*01	peptide	OD1 Asn 71	N Gly 13
DRA1*01/	DRB1*14:04	peptide	OD1 Asn 82	N His 6
DRB1*14:04	DRA1*01	peptide	N Ser 55	O Ala 3
	DRA1*01	peptide	OE2 Glu 57	NE Arg 4
	DRA1*01	peptide	O Ile 65	OG Ser 9
	DRA1*01	peptide	N Val 67	OG Ser 9
	DRA1*01	peptide	O Val 67	NE2 His 12
	DRA1*01	peptide	N Asp 68	O Ala 10
	DRA1*01	peptide	NH1 Arg 78	OG Ser 14
**Salt bridges**				
DRA1*01/	DRA1*01	peptide	OD1 Asp 68	ND1 His 12
DRB1*14:04	DRA1*01	peptide	OD2 Asp 68	ND1 His 12
	DRA1*01	peptide	OD1 Asp 68	NE2 His 12
	DRA1*01	peptide	OD2 Asp 68	NE2 His 12

Zooming out of these three pockets, we should note that other residues formed hydrogen bonds with the peptide. In DRB1*14:04, Asn 82 interacts with His 6, creating a hydrogen bond (Fig 5B, Table 5). Concerning DRB1*11:04 allele, no other H-bonds were found. However, in heterodimer formation, both molecules appear to have closer interactions with the peptide. More specifically, in DRA1*01– DRB1*11:04, the α-chain forms two hydrogen bonds with the peptide. Ser 55 forms two H-bonds with Asp 1 of the peptide while another H-bond is formed between Asn 71 and Gly 13, near the COOH termini of the peptide (Fig 5A, Table 5). DRA1*01 –DRB1*14:04 molecule binds the peptide stronger with a total of 8 hydrogen bonds. One of them is the above-mentioned bond between βAsn 82 and Ser14. DRA1*01 in this heterodimer, forms seven H-bonds in the entire length of the groove. In particular, the following bonds are formed (they are listed in the order they were found from the N-terminus to C-terminus of the peptide) between Ser 55 and Ala 3, Glu 57 and Arg 4, Ile 65 and Ser 9, Val 67 and Ser 9, Asp 68 and Ala 10, Val 67 and His 12 and between Arg 78 and Ser 14 (Fig 5B, Table 4). In addition to these bonds, in DRA1*01 –DRB1*14:04 four salt bridges are also observed between α chain and the peptide. α Asp 68 forms all four ion bonds with peptide’s His 12 (Table 5). No ion bonding was found between heterodimer DRA1*01 –DRB1*11:04 and the peptide nor between DRB1*14:04 and the peptide.

## Discussion

Our previous study revealed an association between MHC class II genes (*HLA-DPA1*, *-DQA1* and -*DRB1*) and WNV infection, using WNV cases and control cohorts. Focusing on the cases, several alleles in all three genes studied were associated with either mild or severe form of the disease supporting a substantial role of MHC class II locus in the progression of WNV disease. In order to understand the different behavior of the alleles (protective or susceptible against severe WNV disease), we brought their amino acid sequences into the spotlight. More specifically, these residues that are located in positions responsible for the antigen recognition and it’s stabilization in the groove; important steps for the TCD4 activation.

The variability of the proteins studied was found according to the literature. HLA-DPA1 was the least polymorphic. In our cases, five alleles were found to produce five different proteins (Fig 2). However, when isolating the PBR four different combinations of P1, P6 and P9 were found. SYFMFWF (P1) and ANILN (P6) were the most frequent variants in both groups. Concerning the least common variants, they also had similar frequencies in both WNND and WNF (Table 1). It seems that none of the alleles found in HLA-DPA1 locus contribute to WNV recognition in different way. Even P6 variant MNISN, that was observed only in WNND (allele DPA1*03:01) was not associated with susceptibility. Residues 50 and 83 were also polymorphic but they don’t belong to the pockets studied. α50 is in the neighbor of the peptide and could probably make contact in some DP-alleles or with certain peptides. However, the frequency of the two amino acids (Gln and Arg) in the two groups discourages any suggestions for possible participation in our case. No structural analysis was performed in the case of HLA-DPA1 due to lack of any trend of association with the severity of the disease.

In the six alleles of HLA-DQA1, five different PBRs were found. These PBRs were the result of the combination between four variants of P1 and P9 and two of P6. All pocket variant were found in both case groups. ACHQFWG (P1), NNVAN (P6) and HNIMR (P9) were the most common variants. ACHQFWG was found with similar frequency in WNND and in WNF cohorts. However, the rest of the variants did not have the same representation in the two groups. Variants AYHEFWR, AYHQFWR, NNVLN, HNIVR, HNILR and HNSLR appeared in the WNF group with almost two times higher (in the case of HNILR three times higher) frequency compared to WNND cases (Table 1). AYHEFWR and NNVLN were the only variants that were found associated with a protective role against WNV neuroinvasion with statistical significance (Fig 3). On the other hand, the frequency of NNVAN (P6) and HNIMR (P9) in the WNND group reached almost 90% in the variants identified in these pockets (Table 1). Thus, both variants were associated with increased risk of neuronal damage. On the contrary to the variable residues found in HLA-DPA1 proteins, in HLA-DQA1 several residues that do not belong in the PBR were polymorphic. Most of them belong in the heterodimerization region between α and β chains (α44–53 region) [26]; more specifically residues 44 (Arg>Gly>Lys>Cys), 45 (Trp>Leu), 47 (Glu>Leu>Val), 48 (Phe>Leu), 49 (Ser>Arg>His), 50 (Lys>Arg>Gln), 51 (Phe>Leu) and 53 (Gly>Arg). This high variability plays a determinant role in α/β chain-pairing that reflects on the variety of peptides that can be recognized by these molecules. Polymorphic residues of the TCR-binding surface such as residues 58 (Gly>Phe) and 61 (Arg>Thr) could affect the TCR-peptide binding and thus the immune response mounted. However, these interactions belong beyond the spectrum of our interest, which is the role of the variable residues in the PBR of HLA class II molecules. Additionally, the lack of data about the β chain in our cases makes any suggestions about the whole structure of the DQ molecule and its interaction with the TCR arguable.

Focusing on the peptide-binding pockets of HLA-DQA1, the two alleles used for comparison show structural differences mostly in P1 and P6. More specifically, the protective variant AYHEFWR carries both more bulkier and charged amino acids compared to ACHQFWG. NNVLN is also more voluminous compared to NNVAN. The increased volume of residues in these pockets compared to DQA1*01:02 may create a shallower groove that could not accommodate peptides with large side chains. In addition, the charges in pocket 1 of DQA1*03:01 define a certain repertoire of peptides that are optimal for anchoring in this pocket creating a peptide-MHC class II stable bond. Increased stability of MHC class II-peptide complex has been associated with immunogenicity [27, 28] as well as with clonotypic diversity of antigen-specific CD4 T cell responses [29]. Moreover, the 18 substitutions that differentiate DQA1*01:02 and DQA1*03:01 could change the structure of the whole groove by influencing the placement and orientation of other residues as well.

For the simulations, we used the available peptide from the crystal structure with PDB ID: 2NNA and not a known virus peptide. For this reason, we did not expect the protective allele to make more interactions with the peptide compared to the susceptible allele. Specifically, we wanted to understand the structural differences between the two alleles and how these differences could influence the peptide binding and not to compare the binding of the peptide (chain D) *per se*. Indeed, we observed that DQA1*01:02/DQB1*03:01 formed more bonds throughout the peptide, stabilizing this specific peptide stronger than DQA1*03:01, that has the protective pocket variants. Although at first glance this was not ideal for making our point, it shows exactly how different these alleles behave towards a given peptide. In Fig 4C we can see how many positions of the groove diverge between the two alleles and in Table 3 we can see how this fact reflects on the peptide binding. One could suggest that DQA1*01:02 and DQA1*03:01 have very different peptide repertoires that could explain the opposite behavior in WNV infection. This hypothesis is confirmed by the recent bibliography on *HLA-DQA1*. More specifically, DQA1*01:02 has been associated with risk for Alzheimer disease [30], pemphigus foliaceus [31] and Parkinson disease [32] and with protection against celiac disease [33] and autoimmune polyglandular syndrome type III [34]. On the other hand, DQA1*03:01 has been associated with susceptibility to pemphigus vulgaris [31, 35], to autoimmune polyglandular syndrome [34] and to type I diabetes [36] and with protection against Parkinson disease [32]. No accordance between the roles of these alleles was observed. On the contrary, in several cases, they presented with opposite effects [32, 34]. Furthermore, when zooming out from the pocket variants, we noticed that in many cases, DQA1*01 presented with opposite association from DQA1*03, *04 and *05. For example in a meta-analysis about the role of MHC class II in pulmonary tuberculosis, DQA1*01:01 was associated with high susceptibility whereas DQA1*04:01 and DQA1*05:01 demonstrated protection against pulmonary tuberculosis [37]. The results of other studies also support this observation [38, 39, 40, 41]. There is no specific pocket variant that is common in DQA1*03, *04 and *05 compared to DQA1*01. However, these alleles can be grouped in two categories based on the amino acid in position 52. Based on our results, Arg-52 (DQA1*03, *04 and *05) is strongly associated with WNF while Glu-52 (DQA1*01) with WNND (*P = 0*.*0001*). Similarly, Leu-66 is associated with WNND and Ala-66 with WNF (*P = 0*.*0001*). We propose that α52 and α66 can be used as markers for risk assessment of neuronic implication during WNV infection.

In β chains more pockets participate in antigen peptide binding thus we focused on six pockets instead of three in α chains. The pocket variability differed between the pockets of HLA-DRB1; the order of polymorphic variants was P1<P6<P10<P9<P4<P7 (min. number of variants 3, max. number of variants 12). These variants were combined, producing the 22 alleles and proteins found in our cases. The most common pocket variants in both cases cohorts were VVFT (P1), SFDDRAY (P4), SY (P6), DFWFR (P7), EYD (P9) and PDY (P10). More specifically, PDY was found in 72/74 of WNF alleles, implying a possible protective role against neuroinvasion. Contrary to the distribution of pocket variants found in HLA-DQA1, several variants were found only in WNND or in WNF group. As shown in Fig 3, ten variants of all pockets were found exclusively in WNND while three variants, belonging in P7, were found only in WNF. Interestingly, none of them was found associated (P>0.005, data not shown) with either of two conditions. The two variants that were found to be associated with a higher risk of CNS involvement were DYWLR in P7 and EFA in P9 (*P = 0*.*02* and *P = 0*.*038* respectively). Apart from the variable residues of the PBR, several other positions were polymorphic. Most of them were close to the pockets we studied and could affect the structure or the charge of the environment around. These positions and the respective amino acids are: 10 (Tyr>Gln>Glu), 31 (Phe>Val), 32 (Tyr>His), 38 (Val>Ala>Leu) and 73 (Ala>Gly). Position 77 that belongs in the TCR binding region was also dimorphic (Thr>Asn). Again, these substitutions were not evaluated since they do not belong in the PBR.

Since only pockets 7, 9 and 10 had variants associated with either WNND or WNF cohort, no other residues were evaluated (if not formed bonds with the peptide, section *Protein modeling*: DRA1*01-DRB1*11:04/DRB1*14:*04*). Concerning the properties of variants DYWLR and EFA that were associated with neuroinvasional symptoms, no amino acid showed any association with the severe form of WNV infection (Table 3). Although in few cases, amino acids in other variants had significantly different characteristics or volumes (for example 71 Arg>Glu>Ala) no specific preference was observed in order to explain the susceptible character of these variants. The combination of DYWLR and EFA was found only in alleles DRB1*14:01 and DRB1*14:04. The frequency of alleles DRB1*14:01 and DRB1*14:04 was significantly higher in WNND group (P = 0.04, data not shown) suggesting a positive association with the severe WNV disease. On the contrary, Asp 57 of the variant PDY was the only charged amino acid found in P10. Since PDY was the abundant variant of this pocket, no association was found suggesting that Asp 57 could contribute to a more effective antigen recognition and immune response. However, the fact that PDY was almost the only variant found in the WNF group may sketch a secondary protective role that should be further explored.

The simulation of the two molecules DRA1*01-DRB1*11:04 and DRA1*01-DRB1*14:04 created two conformations that differed enough structural as well as to the stability of the peptide binding. Different numbers of residues were in immediate vicinity of the peptide in these structures. Zooming in pockets P7, P9 and P10, there were some amino acids whose conformation could affect the selectivity of the binding peptides as DRB1*11:04 Tyr 60 and Phe 67. In addition, different number of residues could interact with the peptide. However, in the case of HLA-DRB1 molecules that resulted from the simulation, the main binding of the peptide is carried from the α chain. Although HLA-DRA1 protein is dimorphic with a monomorphic PBR, the configuration it takes in space when in a complex with another protein can change its role in peptide binding. Again, we used the available peptide from the crystal structure with PDB ID: 2WBJ and not a known virus peptide. However, it is obvious that although HLA-DRA1 is not usually included in the association studies due to lack of polymorphism, in protein level could have a key role. Because of the extreme level of polymorphism of DRB1 locus in combination with our confined number of subjects it was difficult, for a marker-position/pocket variant to raise, as in the case of HLA-DQA1. However, it seems that the presence of either DRB1*14:01 or DRB1*14:04 (alleles with the combination of DYWLR and EFA) increases the risk of a WNV infection to develop to a severe disease. This combination of pocket variants is also found in other 54 DRB1 alleles (all belonging to DRB1*14 group, S1 Table) that should probably be taken into account when encountering with WNV infection.

## Conclusion

In conclusion, seven pocket variants in HLA-DQA1 and HLA-DRB1 molecules were found to have specific roles in WNV infection and further neuroinvasion of the virus. Although a small partition of the alleles of these genes was represented in our cohort, we tried to figure a marker that extends the limits of our study. Indeed, we concluded to a group of alleles of both loci that when found, measures for possible neuronal damage should be taken. Interestingly, none of the pocket variants that were associated with the severe form of the disease was found exclusively in one of the two groups (WNND, WNF). This finding was contrary to our previous results, where protective allele DRB1*11:02 was found only in the WNF group. Further studies about the repertoire of virus peptides that each of these alleles and/or specific pocket variants recognize, should be done in order to understand the character of the optimal peptides for an effective immune response.

## Supporting information

S1 Fig RMSD average and RMSD per residue. A. RMSD average of the four systems DQA1*01:02-DQB1*03:01, DQA1*03:01-DQB1*03, DRA1*01-DRB1*11:04 and DRA1*01-DRB1*14:04. A tendency for structural stabilization can be seen for all four complexes. B. RMSD per residue, chain A. Large deviations from the crystal structure were observed (focused on chain A of DQA1*01:02-DQB1*03:01 and DQA1*03:01-DQB1*03), mainly on flexible parts between secondary structure elements. The pockets under study are not located in these regions. C. RMSD per residue, chain B. Large deviations from the crystal structure were observed (focused on chain B of DRA1*01-DRB1*11:04 and DRA1*01-DRB1*14:04), mainly on flexible parts between secondary structure elements. Other flexible regions that include residues 99–101, 143, 156–157 and 179–180 form beta-sheets but do not form the PBR of the dimer. Some residues that belong to P7, P9 and P10 show both deviation from the crystal structure and different flexibility between the two DR complexes (e.g. regions 35–39 and 57–67 of DRA1*01-DRB1*14:04 and 64–66 of DRA1*01-DRB1*11:04).
(TIF)

S1 Table The DRB1 alleles that carry the pocket variant combination DYWLR/EFA. (DOCX)
