# Supplementary figures and images for: Amino acid signatures in the HLA class II peptide-binding region associated with protection/susceptibility to the severe West Nile Virus disease
Source: PLoS One. 2018 Oct 31;13(10):e0205557. doi: 10.1371/journal.pone.0205557 (PMC6209194; doi:10.1371/journal.pone.0205557)

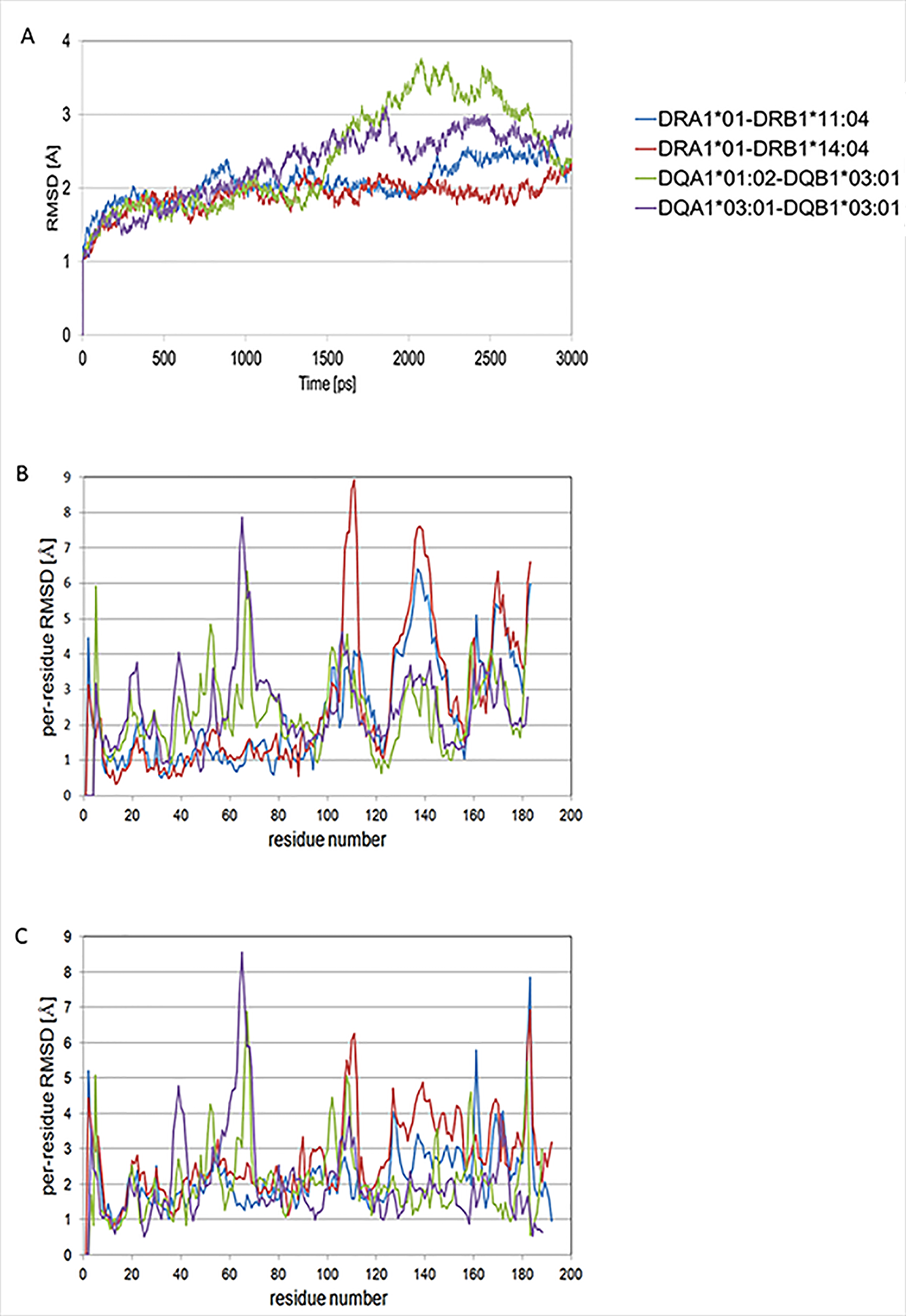

Supplement: S1 Fig — A. RMSD average of the four systems DQA1*01:02-DQB1*03:01, DQA1*03:01-DQB1*03, DRA1*01-DRB1*11:04 and DRA1*01-DRB1*14:04. A tendency for structural stabilization can be seen for all four complexes. B. RMSD per residue, chain A. Large deviations from the crystal structure were observed (focused on chain A of DQA1*01:02-DQB1*03:01 and DQA1*03:01-DQB1*03), mainly on flexible parts between secondary structure elements. The pockets under study are not located in these regions. C. RMSD per residue, chain B. Large deviations from the crystal structure were observed (focused on chain B of DRA1*01-DRB1*11:04 and DRA1*01-DRB1*14:04), mainly on flexible parts between secondary structure elements. Other flexible regions that include residues 99–101, 143, 156–157 and 179–180 form beta-sheets but do not form the PBR of the dimer. Some residues that belong to P7, P9 and P10 show both deviation from the crystal structure and different flexibility between the two DR complexes (e.g. regions 35–39 and 57–67 of DRA1*01-DRB1*14:04 and 64–66 of DRA1*01-DRB1*11:04). (TIF) [file pone.0205557.s001.tif]
