# Supplementary material for: Amino acid signatures in the HLA class II peptide-binding region associated with protection/susceptibility to the severe West Nile Virus disease
Source: PLoS One. 2018 Oct 31;13(10):e0205557. doi: 10.1371/journal.pone.0205557 (PMC6209194; doi:10.1371/journal.pone.0205557)
Supplement: S1 Table — (DOCX) [file pone.0205557.s002.docx]

| HLA-DRB1 alleles | | | |
| --- | --- | --- | --- |
| DRB1*14:01 | DRB1*14:61 | DRB1*14:104 | DRB1*14:143 |
| DRB1*14:04 | DRB1*14:68 | DRB1*14:110 | DRB1*14:145 |
| DRB1*14:07 | DRB1*14:70 | DRB1*14:112 | DRB1*14:146 |
| DRB1*14:10 | DRB1*14:71 | DRB1*14:113 | DRB1*14:147 |
| DRB1*14:26 | DRB1*14:74 | DRB1*14:114 | DRB1*14:148 |
| DRB1*14:28 | DRB1*14:75 | DRB1*14:117 | DRB1*14:149 |
| DRB1*14:31 | DRB1*14:82 | DRB1*14:118 | DRB1*14:150 |
| DRB1*14:32 | DRB1*14:86 | DRB1*14:119 | DRB1*14:151 |
| DRB1*14:38 | DRB1*14:87 | DRB1*14:125 | DRB1*14:153 |
| DRB1*14:39 | DRB1*14:88 | DRB1*14:129 | DRB1*14:157 |
| DRB1*14:50 | DRB1*14:90 | DRB1*14:131 | DRB1*14:158 |
| DRB1*14:54 | DRB1*14:93 | DRB1*14:139 | DRB1*14:163 |
| DRB1*14:55 | DRB1*14:99 | DRB1*14:140 | DRB1*14:164 |
| DRB1*14:60 | DRB1*14:101 | DRB1*14:142 | DRB1*14:168 |

**S1 Table.** The DRB1 alleles that carry the pocket variant combination DYWLR/EFA.
